# Lemierre's Syndrome: A Case Report of Thrombophlebitis of the Internal Jugular Vein Induced by Laryngeal Cancer

**DOI:** 10.7759/cureus.65060

**Published:** 2024-07-21

**Authors:** José Manuel García Romero, Fatima Paulina Jaime Vargas, José Gonzalo Bravo Quiroz, Diana Pérez Mondragón, Pablo Hernández Guillen

**Affiliations:** 1 Transplant and Donation Department, Regional General Hospital 1 of the Mexican Institute of Social Security, Querétaro, MEX; 2 General Practice Department, Centro de Salud Pedro Escobedo, Secretaría de Salud, Querétaro, MEX; 3 General Practice Department, Centro de Salud San Pedrito Peñuelas, Secretaría de Salud, Querétaro, MEX

**Keywords:** fusobacterium necrophorum, laryngeal cancer, lemierre's syndrome, jugular vein thrombosis, septic thrombophlebitis

## Abstract

Lemierre's syndrome, a rare complication of oropharyngeal infections, is characterized by septic thrombophlebitis primarily affecting the internal jugular vein, which can lead to septicemia, thrombotic obstruction, and potential dissemination to distant sites. We present the case of a 54-year-old male with a history of chronic smoking and newly diagnosed laryngeal carcinoma, complicated by Lemierre's syndrome. Initial symptoms included odynophagia, dyspnea, and cervical swelling, with subsequent diagnosis confirming thrombosis of the internal jugular vein via ultrasound, CT, and MRI. Treatment included broad-spectrum antibiotics and anticoagulation, followed by oncological management for the carcinoma. This case underscores the diagnostic challenge posed by concurrent malignancy and highlights the critical role of early recognition and comprehensive treatment involving antibiotics, anticoagulation, and oncological therapy. Multidisciplinary collaboration is crucial for optimizing outcomes in complex cases like this, emphasizing the need for heightened clinical suspicion and prompt intervention involving proper imaging diagnosis, appropriate antibiotic coverage, and timely microbiological recognition for adjustment of antimicrobial therapy.

## Introduction

Lemierre's syndrome, also known as post-anginal sepsis or suppurative jugular thrombophlebitis, is a rare condition first described by André Alfred Lemierre in 1936 [1,2]. It typically arises as a complication of an oropharyngeal infection, leading to sepsis and septic emboli. The incidence of the disease is reported to be 14.4 cases per million per year in the population aged 15-24 years old, and it is rare in adults over 40 years of age, with an incidence of 1.4 cases per million per year [3].

Clinically, it manifests with a sudden onset of systemic illness characterized by symptoms such as chills, tremors, and high fever, often accompanied by profound weakness, dysphagia, dysphonia, jaw angle pain, inflammation of the peritonsillar region, and unilateral neck swelling [4]. The syndrome is further complicated by septicemia, primarily caused by *Fusobacterium* species, anaerobic gram-negative bacilli commonly found in the normal oral flora. This leads to the formation of blood clots in the internal jugular vein, with *Fusobacterium necrophorum* responsible for over 80% of cases, making it the predominant pathogen associated with the condition [4,5]. 

Diagnosis requires demonstrating thrombotic obstruction specifically within the jugular veins, along with confirming bacterial presence through a positive culture. This approach ensures the identification of direct jugular vein involvement in the thrombotic process and verifies the microbial cause, which is crucial for accurate diagnosis and appropriate treatment planning [2]. The infection can originate from perivascular extension, septic emboli from peritonsillar veins, lymphatic spread, or contiguous spread [6]. These conditions enable anaerobic bacteria to invade surrounding tissues, often involving the jugular veins. The most common sites of contiguous spread or hematogenous dissemination of septic emboli are the lungs (79.8%) and joints (16.5%), which can lead to pneumonia and septic arthritis [7]. While most cases of Lemierre's syndrome stem from naso-/oropharyngeal infections, sinusitis, otitis, or chest infections, there are discussions regarding the presence of cancer as an associated factor in the development of Lemierre's syndrome in specific cases [3].

## Case presentation

A 54-year-old male patient, with a history of chronic smoking (20 pack-years) and moderate alcohol consumption, presented with a two-week history of persistent odynophagia, dyspnea, progressive dysphonia, fever, and general malaise. He also reported recurrent upper respiratory tract infections and unexpected recent weight loss. Physical examination revealed pharyngeal erythema and cervical edema. A laryngoscopy showed an ulcerated lesion on the left vocal cord. Additionally, a palpable mass was observed in the left cervical region (zone 3 of the neck), which was nodular, was slightly mobile, and had an indurated consistency but was not adhered to deep planes.

**Figure 1:**
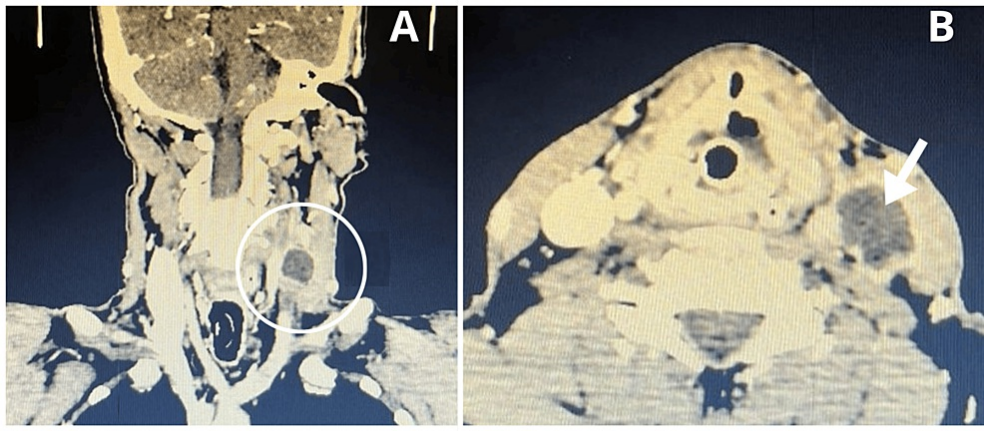
Head CT CT images with contrast in (A) coronal and (B) axial reconstructions demonstrate a hypodense lesion located in the internal jugular vein (white circle), characterized by well-defined borders, an oval-shaped morphology, and a hyperdense periphery (white arrow) suggestive of luminal thrombus occupation in the left internal jugular vein.

Laboratory analysis revealed a white blood cell count of 22,000/μl, prompting blood cultures which subsequently reported positive for *Fusobacterium nucleatum* after five days. Broad-spectrum antibiotic therapy with ceftriaxone and metronidazole was initiated. Ultrasound identified a well-defined heterogeneous nodular occupational lesion measuring 27×22×15 mm, with vascularity noted on color Doppler consistent with thrombophlebitis. The lesion completely obstructed the left internal jugular vein and partially compressed the left carotid artery. A non-contrast CT scan confirmed a mass obstructing the course of the left common carotid artery (Figure 1).

**Figure 2:**
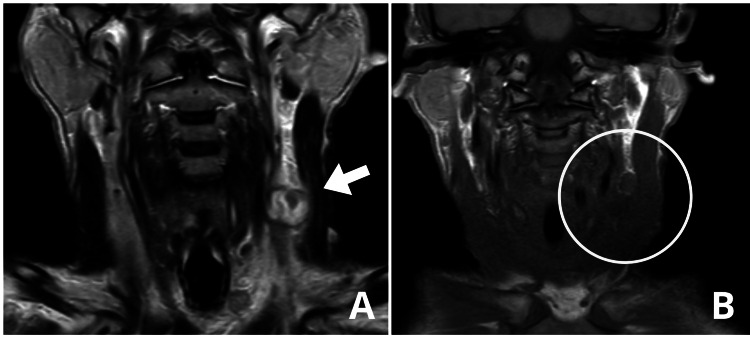
MRI of the head MRI with contrast in T1-weighted sequence (A) shows an oval-shaped image with well-defined borders (white arrow), located in the region of the left internal jugular vein, consistent with thrombophlebitis. MRI with contrast in T2-weighted sequence (B) displays a heterogeneous image predominantly hyperintense with a hypointense peripheral halo, measuring approximately 2.4×2.1 cm (white circle).

Due to the involvement of neck vessels, a contrast MRI was performed, confirming the total obstruction of the left internal jugular vein, indicative of Lemierre's syndrome (Figure 2).

After initial treatment for septic shock, involving antibiotics targeting aerobic and anaerobic bacteria, the patient underwent a tracheostomy due to prolonged intubation. An incisional biopsy of the cervical mass yielded two irregular tissue fragments with an anfractuous surface, totaling 1×0.5×0.5 cm in size and whitish-brown in color, which were sent for histopathological analysis. Additionally, six fragments of peritracheal tissue were included, measuring a combined volume of 4×1×0.5 cm, with a solid cut surface and similar coloration. The analysis identified well-differentiated invasive squamous cell carcinoma of the larynx with invasion into the peritracheal tissue. Additionally, invasion into soft tissues of the cervical region and infiltration into the perichondrium of the tracheal ring were noted. Lymphovascular invasion was present, while residual thyroid tissue showed no signs of infiltration.

Treatment was initiated with chemotherapy and radiotherapy. Upon discharge, the patient underwent four weeks of broad-spectrum antibiotics and received heparin for anticoagulation. He responded well, with the resolution of fever and improvement in neck pain. Oral anticoagulation was continued as outpatient therapy.

## Discussion

Septic thrombophlebitis of the jugular veins is a rare complication that should be considered when evaluating any lateral cervical mass presenting with systemic signs of sepsis and a history of oropharyngeal infections [6]. In this case, the patient's history of smoking was a significant risk factor for laryngeal cancer, contributing to hypercoagulability, inflammation, and blood stasis leading to thrombophlebitis.

Mortality from Lemierre's syndrome has decreased from 90% in the pre-antibiotic era to 4-10% currently. However, it continues to cause significant morbidity due to its elusive nature and the increasing prevalence of antibiotic resistance [8]. Due to the sparse areolar connective tissue within the carotid sheath in this area, there is a low tendency for upward or downward spread within the vascular sheath, except for retrograde thrombophlebitis and potential intracranial extension [1].

In recent years, several authors have noted an increase in the number of cases of Lemierre's syndrome. This trend could be attributed to several factors, including restrictions in antibiotic prescriptions for treating pharyngitis, increased use of macrolides (which are ineffective against *F. necrophorum*), and reduced indications for tonsillectomies [9]. Changes in antibiotic use, such as overuse or misuse, may contribute to alterations in bacterial populations, potentially favoring the emergence of resistant strains. This shift could lead to greater challenges in effectively treating infections, including those associated with this particular disease. The preferred therapy for Lemierre's syndrome involves a beta-lactam antibiotic combined with metronidazole for a duration of 2-6 weeks, as there is growing resistance to penicillin. Due to the presence of beta-lactamase-producing strains of *F. necrophorum*, treatment options should include piperacillin-tazobactam, carbapenems, or ceftriaxone with metronidazole. The duration of treatment may need to be extended based on the severity of the illness [10].

Anticoagulation is specifically recommended for patients with delayed initiation of adequate antimicrobial therapy, persistent bacteremia, underlying thrombophilia, or identified intracranial thrombosis [8]. Therefore, management trends lean towards using low-molecular-weight heparin in critically ill patients, particularly when there is a poor response to antibiotic therapy, although consensus on the duration of anticoagulation remains unsettled [11].

Most cases of suppurative jugular thrombophlebitis can be managed medically without the necessity for ligature or surgical resection of the infected vein. In the past, a surgical technique during the era of Lemierre involved ligating the internal jugular veins, a practice seldom employed in contemporary medicine except in cases of persistent septic embolization or cases requiring drainage of abscesses or collections [3,8].

## Conclusions

This case underscores the critical importance of considering Lemierre's syndrome in patients presenting with persistent oropharyngeal infections, particularly those with predisposing factors such as malignant neoplasms. Smoking, a significant risk factor for laryngeal cancer, underscores the heightened risk of developing conditions like Lemierre's syndrome. This syndrome can arise secondary to neck cancers, highlighting the need for heightened vigilance in these patients. A multidisciplinary approach involving oncologists and infectious disease specialists is essential for comprehensive and successful management. Timely initiation of antibiotics and anticoagulation is paramount for improving the prognosis of these patients, emphasizing the complexity and urgency of managing such cases.
